# Atypical Presentation of Creutzfeldt-Jakob Disease: A Stroke Mimic

**DOI:** 10.7759/cureus.100231

**Published:** 2025-12-28

**Authors:** Sadia Faisal, Zoya Malik, Samaah Fathima, Abdul Salam, Ragunath Durairajan

**Affiliations:** 1 Stroke Medicine, Russells Hall Hospital, Dudley, GBR; 2 Medicine, Nottingham City Hospital, Nottingham, GBR; 3 Internal Medicine, Russells Hall Hospital, Dudley, GBR

**Keywords:** cjd, csf, prion disease, rt-quic, stroke mimic

## Abstract

A 56-year-old woman presented with a five-week history of unsteadiness, progressive diplopia, tremulous speech, and mild cognitive decline. Although initial computed tomography (CT) imaging was normal, magnetic resonance imaging (MRI) revealed bilateral diffusion restriction in the basal ganglia and thalami, raising suspicion for prion disease, which was subsequently confirmed by cerebrospinal fluid real-time quaking-induced conversion (CSF RT-QuIC) testing. A neurological examination showed saccadic intrusions, persistent horizontal diplopia, mild limb hypertonia, right-sided dysmetria, absent ankle reflexes, and ataxic gait, while extensive metabolic, autoimmune, and toxicology evaluations were unremarkable and family history was noncontributory. This atypical presentation, initially dominated by cerebellar and ocular-motor features, highlights the diagnostic challenges of early Creutzfeldt-Jakob disease (CJD) and the value of advanced neuroimaging and specialized assays, as well as the importance of coordinated multidisciplinary care following diagnosis.

## Introduction

Creutzfeldt-Jakob disease (CJD) is a rare, fatal neurodegenerative disorder caused by misfolded prion proteins. It is part of the group of transmissible spongiform encephalopathies that occur worldwide. Diagnosis can be particularly challenging when patients present with atypical features [1]. The majority of Creutzfeldt-Jakob disease cases are sporadic type (sCJD), making up about 85-90%, while the remaining cases are familial, iatrogenic, or variant forms. The average age of onset for sCJD is around 65, with most patients falling between 60 and 80 years old. It’s unusual to see sCJD in individuals younger than 30 or older than 80 years. The exact cause remains unclear, and it affects men and women at roughly the same rate [2].

The present case describes a 56-year-old woman whose initial symptoms were dominated by gait unsteadiness, diplopia, tremulous speech, and mild cognitive decline. The predominance of cerebellar and ocular-motor signs made the diagnosis less clear in the early phase. Her course underscores the difficulty of recognizing atypical presentations of CJD and highlights the importance of appropriate neuroimaging and CSF testing in patients with unexplained, rapidly progressive neurological symptoms.

## Case presentation

**Figure 1:**
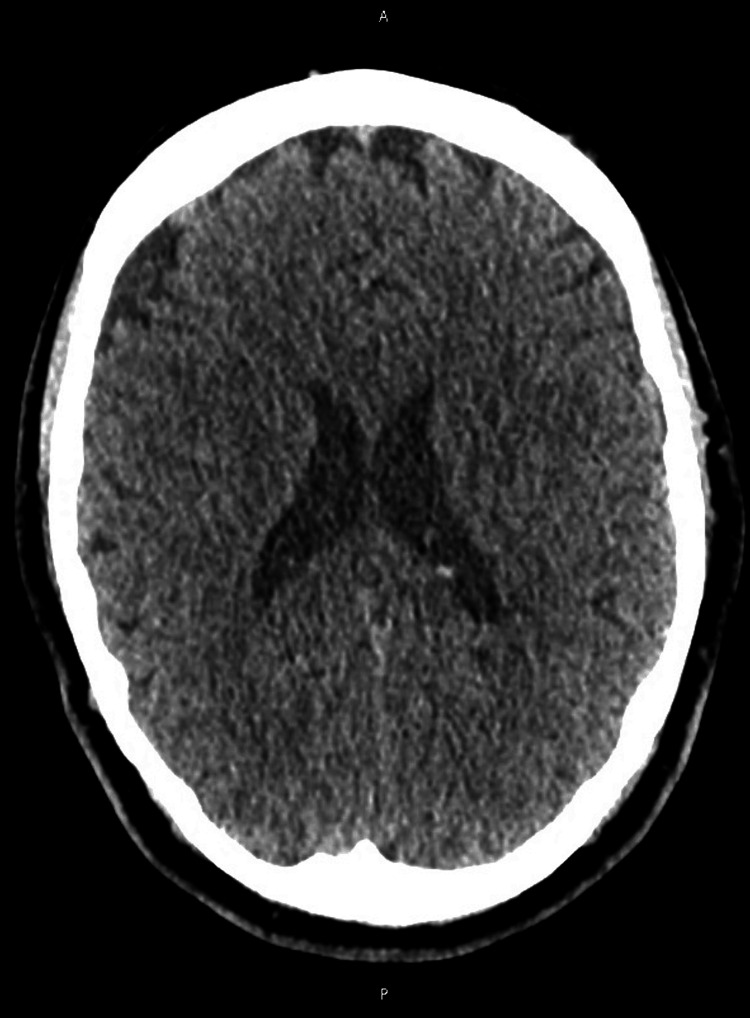
Computed tomography (CT) image of the brain No evidence of acute intracranial abnormality is visible.

**Figure 2:**
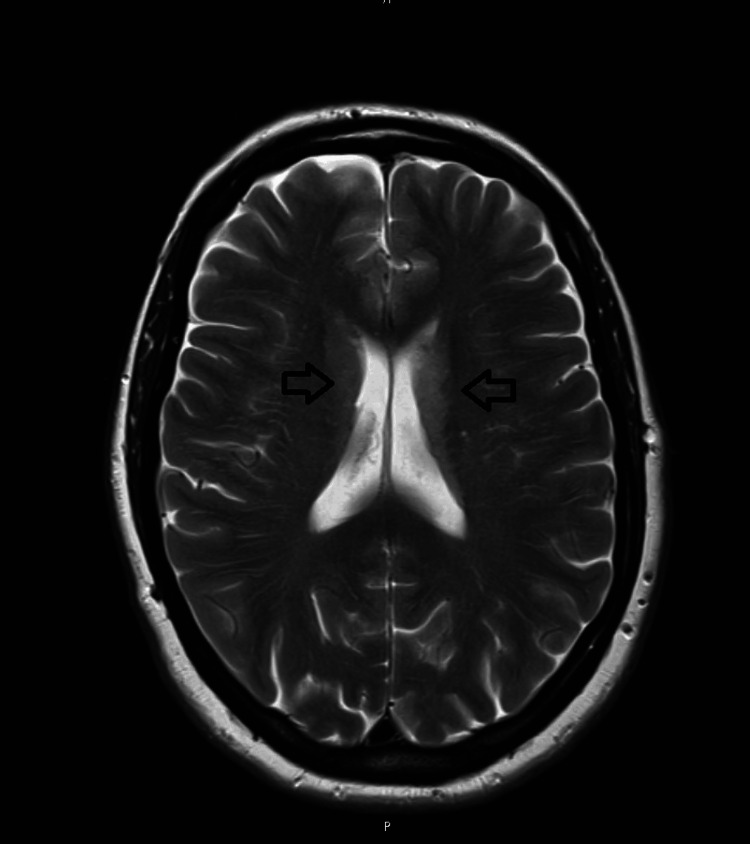
Magnetic resonance imaging (MRI) image of the brain Abnormal diffusion restriction in the basal ganglia and bilateral thalami as described is concerning; differential includes Creutzfeldt–Jakob disease (CJD), recent seizure activity, and previous hypoxic/metabolic encephalopathy.

**Figure 3:**
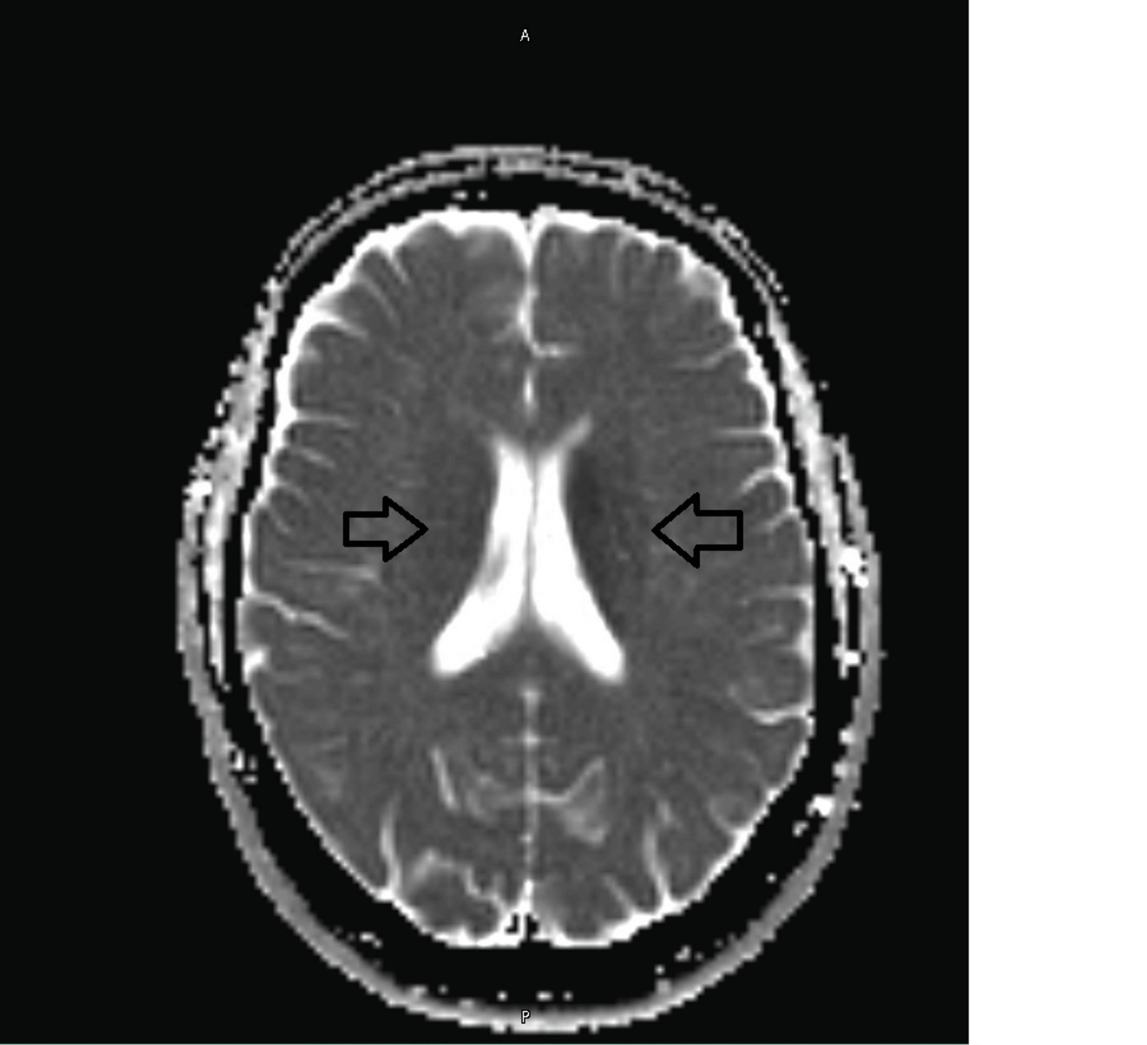
Magnetic Resonance Imaging (MRI) ADC image ADC: Apparent Diffusion Coefficient. Abnormal diffusion restriction in the basal ganglia and bilateral thalami, as described, is concerning; differential includes CJD, recent seizure activity and previous hypoxic/metabolic encephalopathy.

A 56-year-old female patient presented with a five-week history of unsteadiness, progressively worsening double vision, unsteady handwriting, and subtle cognitive decline with mild forgetfulness. She also had an intermittent dry cough. A computed tomography (CT) head scan was unremarkable (Figure 1). However, the subsequent magnetic resonance imaging (MRI) revealed bilateral diffusion restriction in the basal ganglia and thalami, more pronounced on the left (Figures 2, 3). This raised suspicion for prion disease and prompted consideration of Creutzfeldt-Jakob disease (CJD) in the differential diagnosis. A cerebrospinal fluid (CSF) real-time quaking-induced conversion (RT-QuIC) test confirmed the diagnosis of CJD.

Her past medical history was significant only for hypertension. Extensive investigations, including neuroimmunology screening, serum immunoglobulin levels, and urine drug screening, were negative. Family history was non-significant for neurodegenerative disease. On cognitive assessment, the patient scored 27/30 on the Mini-Mental State Examination (MMSE) and 16/20 on the Medical Research Council (MRC) cognitive scale. Neurological examination revealed saccadic intrusions during smooth pursuit, which were hypometric in all directions. The patient had horizontal diplopia in all directions of gaze, more pronounced when looking to the left. This did not resolve with monocular occlusion or head tilt. Her speech was slightly tremulous, although there was no evidence of dysarthria. Muscle tone was mildly increased in the right leg, and muscle strength was preserved throughout. Mild dysmetria was noted on the finger-to-nose test on the right side, with otherwise intact coordination. Ankle reflexes were absent, and all other reflexes were present. The patient was unable to perform tandem walking and had an ataxic gait, and he required support from one person to mobilise.

The patient was reviewed by the teams at the National Prion Clinic and the National CJD Surveillance Unit, both of whom confirmed the diagnosis based on her clinical features, Magnetic Resonance Imaging (MRI) of the head, and CSF RT-QuIC. Following confirmation of diagnosis, the patient received community-based rehabilitation alongside palliative support and was subsequently discharged home. 

This case report illustrates an atypical presentation of CJD. Patients presenting with cerebellar signs with other atypical features should have CJD considered in their differential diagnoses. This case also highlights the challenges in the early diagnosis of CJD, especially when initial symptoms are subtle. Advanced neuroimaging and specialized diagnostic tools such as the RT-QuIC assay are essential for accurate and timely diagnosis.

## Discussion

Creutzfeldt-Jakob disease (CJD) is a rare, currently untreatable disorder with an annual global incidence of about one to two cases per million. It occurs in sporadic, familial, iatrogenic, and variant forms [3,4]. In this case, the patient was diagnosed with the sporadic type. Early diagnosis of sCJD and other prion diseases is often difficult because of the rapid disease progression and wide clinical variability. Patients may present with progressive, multifocal neurological signs and symptoms, such as rapidly progressive cognitive decline with dementia, visual disturbances, movement disorders, myoclonus, and ataxia, many of which overlap with other neurological conditions [5]. Additionally, atypical clinical presentations, including prolonged disease courses in some sCJD cases or rare genetic forms, further complicate timely and accurate diagnosis.

The phenotypic variants of sporadic CJD present with distinct early features. The amyotrophic variant begins with symptoms that resemble amyotrophic lateral sclerosis. The Brownell-Oppenheimer variant starts with cerebellar ataxia. The Heidenhain variant is marked by early visual problems, including reduced visual acuity, distorted shapes and colours, and visual hallucinations. The Stern Garcin variant begins with extrapyramidal symptoms [6].

Pre-mortem diagnostic tools for prion disease include Magnetic Resonance Imaging (MRI), electroencephalogram (EEG), cerebrospinal fluid (CSF) analysis, and blood tests to exclude alternative conditions. Brain MRI, especially diffusion-weighted imaging, offers very high sensitivity and specificity for Creutzfeldt-Jakob disease (CJD). Similarly, the real-time quaking-induced conversion (RT-QuIC) assay on CSF is highly accurate. At present, there is no cure for prion diseases. Management focuses on symptom relief and palliative care. In sporadic prion disease, most patients survive for about a year or less following symptom onset. Survival in genetic prion diseases varies widely, ranging from several months to several years, depending on the specific mutation involved. [7]

This case adds to the growing recognition that CJD may first appear as an unexplained cerebellar syndrome with ocular-motor abnormalities, even when cognitive findings are mild. For clinicians, the key lesson is to consider CJD early when evaluating patients with rapid functional decline, cerebellar signs, or progressive visual symptoms without an alternative explanation. Early diagnosis supports timely counselling, coordinated multidisciplinary management, and appropriate palliative planning for patients and families.

## Conclusions

The patient was discharged home with comprehensive community support, involving relevant multidisciplinary teams including therapists, the palliative care team, and a neurologist. At follow-up with these services, her condition was noted to have progressed, with increasing mobility impairment requiring the use of a walking stick and worsening cognitive symptoms, particularly forgetfulness. This case highlights an atypical presentation of Creutzfeldt-Jakob disease, initially dominated by cerebellar and oculomotor features that obscured the underlying diagnosis. Subtle early symptoms, unremarkable initial imaging, and a broad differential diagnosis for gait and visual disturbances contributed to a delay in recognition. Ultimately, advanced MRI sequences and cerebrospinal fluid RT-QuIC testing were pivotal in confirming the diagnosis, underscoring their critical role in the evaluation of rapidly progressive neurological syndromes. Early identification of CJD allows for timely patient and family counselling, coordinated multidisciplinary management, and appropriate palliative care planning. As demonstrated by this case, clinicians should maintain a high index of suspicion for CJD in patients presenting with unexplained cerebellar signs and rapid functional decline to ensure that rare but devastating conditions are not overlooked.
